# A composite nanocarrier to inhibit precipitation of the weakly basic drug in the gastrointestinal tract

**DOI:** 10.1080/10717544.2020.1760402

**Published:** 2020-05-13

**Authors:** Chunli Zheng, Yun Li, Zhen Peng, Xinyi He, Juan Tao, Liang Ge, Yixin Sun, Yunkai Wu

**Affiliations:** a Department of Pharmaceutics, China Pharmaceutical University, Nanjing, China;; b School of Pharmacy and Traditional Chinese Pharmacy, Jiangsu College of Nursing, Huaian, China;; c Children’s Hospital of Nanjing Medical University, Nanjing, China;; d School of Basic Medical Sciences, Anhui Medical University, Hefei, China

**Keywords:** Weakly basic drug, drug precipitation, drug leakage, P-glycoprotein inhibitor, composite nanocarrier

## Abstract

For weakly basic drugs, the sharp decrease of drug solubility and the following drug precipitation after drugs transferring from the gastric fluid to the intestinal fluid in the gastrointestinal (GI) tract is a main reason for the poor oral bioavailability of drugs. Here, an anticoagulant dabigatran etexilate (DE) was used as a model drug, and a composite nanocarrier system of DE was developed to improve the drug dissolution by decreasing the drug leakage in the stomach and inhibiting the drug precipitation in the intestinal tract. With the encapsulation of drugs in nanocarriers, the precipitation percentage of DE in composite nanocarriers was 22.25 ± 3.88% in simulated intestinal fluid, which was far below that of the commercial formulation. Moreover, the relative bioavailability of DE-loaded composite nanocarriers (456.58%) was greatly enhanced and the peak of its activated partial thromboplastin time was also significantly prolonged (*p <* .01) compared with the commercial formulation, indicating that the anticoagulant effect of DE was effectively improved. Therefore, the designed composite nanocarrier system of DE presents great potentials in improving the therapeutic efficiency and expanding the clinical applications of poorly water-soluble weakly basic drugs.

## Introduction

A low solubility of drugs is an important determinant of the poor absorption in the gastrointestinal (GI) tract and insufficient bioavailability *in vivo* (Serajuddin, 2007). Weakly basic drugs usually keep the ionized form and dissolved in the acidic environment, but part of dissolved drugs could precipitate after the medium transferring from the acidic environment to the basic environment (Gu et al., 2005; Dai, 2010). As the pH in the GI tract varies widely with location, a significant changes in solubility of weakly basic drugs will happen during gastrointestinal passage, which could lead to the poor oral bioavailability and thus restrict the clinical applications of drugs (Kostewicz et al., 2004).

Several approaches have been employed to decrease the precipitation of weakly basic drugs in the GI tract, such as the physical mixtures of acidic excipients with drugs, amorphous solid dispersions and the usage of salt forms of drugs (Tatavarti & Hoag, 2006; Williams et al., 2013; Rubbens et al., 2016). Parikh et al. developed an amorphous solid dispersion of itraconazole, which improved the drug solubility in the high pH medium (Parikh et al., 2016). In their study, the physical state of itraconazole was converted from crystalline to amorphous form with higher Gibbs free energy upon drying and preparing solid dispersions. However, amorphous solid dispersion is unstable because the amorphous state of drugs could convert back to the energetically more favorable crystalline state, and the formed small crystals could aggregate into drug precipitation (Leuner & Dressman, 2000; Craig, 2002). In addition, Chai et al. found that the simple mixture of acidic excipients with drugs or the usage of salt forms of drugs such as Pradaxa^®^ capsules could only improve the drug dissolution due to the increased solubility of drugs in the stomach, but most of the drugs dissolved in the gastric fluid still precipitated in the intestinal fluid after oral administration (Chai et al., 2016).

Nanocarriers including inorganic nanoparticles, liposomes, nanoemulsions, and polymeric micelles have been reported to be promising drug delivery vehicles for poorly water-soluble drugs to increase the drug solubility (Van Speybroeck et al., 2010; Guo et al., 2016; Quan et al., 2017; Suys et al., 2018). Unfortunately, the effect of these nanocarriers on inhibiting the precipitation of weakly basic drugs *in vivo* has not been deeply studied. In our previous work, a dabigatran etexilate-loaded solid self-nanoemulsifying system and a phospholipid complex nanoemulsion system of dabigatran etexilate were designed to decrease the precipitation of the drug in the GI tract (Chai et al., 2016; Ge et al., 2017). Both systems resulted in remarkable decreases in drug precipitation and obvious improvements in oral bioavailability compared with the commercial formulation due to the encapsulation of DE into an oil phase of nanoemulsions. However, a large number of surfactants used as nanoemulsions would stimulate the GI tract, thus causing an increased incidence of GI side effects (Poelma et al., 1991; Wignot et al., 2004). Jin et al. prepared a micelle system for a weakly basic drug asulacrine, which prevented drugs precipitating in vein with drugs wrapped in the hydrophobic core of micelles, and pharmacokinetic studies demonstrated that the half-life and AUC values of asulacrine-loaded micelles were approximately 1.37-fold and 3.49-fold greater than that of free drugs (Jin et al., 2018). Thus, micelles showed great potential in improving the *in vitro* dissolution and enhancing the bioavailability of weakly basic drugs. In recent years, composite micelles composed of different polymers attracted increasing attention due to the more functions of composite micelles compared with that of single micelles. For example, a doxorubicin-loaded Tetronic T1107/TPGS mixed micelle system prepared by Cagel et al. exhibited a higher drug release in an acidic microenvironment because of the pH-dependent nature of Tetronic T1107 and enhanced cytotoxic activity due to the selective anti-cancer effect of TPGS (Cagel et al., 2017). Duan et al. prepared curcumin-loaded mPEG-PLA/TPGS mixed micelles, and the P-gp efflux system was inhibited with TPGS by influencing the activity of P-gp ATPase (Dabholkar et al., 2006; Mu & Seow, 2006; Duan et al., 2016).

Dabigatran etexilate (DE) is a novel, reversible and direct thrombin inhibitor belonging to the BSC class II, and it possesses a higher clinical efficacy than conventional anticoagulants (Bellamy et al., 2009; Paikin et al., 2011; Pollak and McBane, 2014; Greig & Mckeage, 2014). Pradaxa^®^ is the commercial formulation of DE, which has been marketed by the US Food and Drug Administration in 2014 for prevention in patients with nonvalvular atrial fibrillation and has been granted by the European Medicines Agency in the same year for treatment of thromboembolic disease following elective total hip or knee replacement surgery(Härtter et al., 2012a,b; Greig & Mckeage, 2014). It has been employed as an alternative to warfarin in clinical therapy due to a quickly absorption and the stable hematologic response after oral administration (Ganetsky et al., 2011). However, the absolute bioavailability of DE is very low (approximately 6.5%) after oral administration, thus restricts the clinical applications of the drug (Greig & Mckeage, 2014). Other studies also have found that DE shows a moderate affinity to the energy-dependent efflux transporter P-glycoprotein (P-gp), which actively pumps drugs out of enterocytes and decrease the amount of drugs for absorption in the GI tract (Makhey et al., 1998; Mundada & Sawant, 2018).

In this study, DE was used as a model drug and dabigatran etexilate-loaded composite micelles (DE-CMs) were developed to decrease drug precipitation in the GI tract. *In vitro* leakage studies were conducted in SGF, SIF and distilled water to investigate the leakage of DE in media with different pH value. *In vitro* precipitation studies were conducted in the simulated gastrointestinal fluid to mimic the real conditions of DE during gastrointestinal passage *in vivo*. The P-gp inhibitor was used in nanocarriers and a Caco-2 cell monolayer model was established to investigate the inhibition of DE-CMs on P-gp efflux with an apparent permeability coefficient (Papp) value and efflux ratio (ER) value. Finally, pharmacokinetic and pharmacodynamic studies were performed in rats to evaluate the effects of DE-CMs on bioavailability and anticoagulation activity of DE.

## Materials and methods

### Materials and animals

Dabigatran etexilate was obtained from Changzhou Yuanda Pharmaceutical Chemical Co., Ltd. (Changzhou, China). Soluplus was generously provided by BASF Corp (Germany). TPGS was purchased from Aladdin Bio-Chem Technology Co., Ltd. (Shanghai, China). Commercial formulation of dabigatran etexilate Pradaxa^®^ capsules were supplied by Boehringer Ingelheim (Germany). DMEM (high glucose) was purchased from GIBCO Co., Ltd. (America). FBS was purchased from ExCell Biology Inc. (China, shanghai). Penicillin/streptomycin solution were provided by KeyGEN BioTECH Co., Ltd. (Nanjing, China). All other chemicals and solvents were of analytical grade or better.

Male Sprague-Dawley rats (SPF grade) weighing 200 ± 20 g were provided by the Animal Center of Qinglong Mountain (Nanjing, China). All studies were conducted under the guidelines approved by the ethics committee of China Pharmaceutical University.

### Preparation of single micelles and composite micelles

Dabigatran etexilate-loaded composite micelles (DE-CMs) were prepared by a membrane hydration method (Yan et al., 2016). Briefly, a ratio of soluplus and TPGS of 7:1 (the total amount is 200 mg) was mixed with 13.71 mg of DE. The mixture was dissolved in 4 mL of methanol, and then the solvent was removed by rotary evaporation at 37 °C for 1 h to form a thin film. Finally, the thin film was kept in a vacuum oven overnight and hydrated with 5.2 mL of deionized water to obtain the DE-CMs. Dabigatran etexilate-loaded single micelles (DE-SMs) were also prepared by a membrane hydration method. Briefly, 13.71 mg of DE and 200 mg of soluplus was dissolved in 4 mL of methanol, then the mixture was treated as described above.

### Characterization of DE-CMs

The mean particle size and the polydispersity index (PDI) of DE-CMs were determined by dynamic light scattering (DLS) measurements using a Zetasizer Nano ZS (Malvern, Worcestershire, UK). PDI was used to describe the width of the particle size distribution and it was calculated from a Cumulants analysis of the DLS measured intensity autocorrelation function (Zanetti-Ramos et al., 2006). A transmission electron microscope (TEM, H-7650, Hitachi, Tokyo, Japan) was used to observe the morphology of DE-CMs.

Differential scanning calorimetry (DSC) and X-ray powder diffractometry (XRD) were employed to analyze the phase state of DE in DE-CMs and to further confirm the formation of DE-CMs (Liu et al., 2012). The thermal properties of samples were investigated using a DSC (DSC-1, Mettler-Toledo, Switzerland) with the scanning temperature set from 40 to 300 °C at a heating rate of 10 °C/min under the dry nitrogen stream. The XRD patterns of samples were measured by an X-ray diffractometer (D8 Advance, Bruker AXS) with Cu-Kα line as the source of radiation. The tube voltage and current were set to 40 kV and 40 mA, respectively, and the 2*θ* angle ranged from 3° to 40°.

### Encapsulation efficiency (EE) of DE-CMs

Encapsulation efficiency (EE) was performed by an ultrafiltration method (Zhong et al., 2017). Briefly, 0.5 mL of DE-CMs was placed into an ultracentrifuge tube (10 kDa) and centrifuged at 4000 rpm for 30 min. Then, the filtrate was collected and analyzed by a UV spectrophotometer (UV-8453, Agilent Technologies, USA) at 316 nm to determine the content of free drugs not encapsulated in micelles. The total content of drugs fed into DE-CMs was also measured by the UV method after micelles were demulsified by ethanol. EE was calculated based on the following equations:

(1) $$\text{EE}\;(\%)=\frac{C_{\text{total}}-C_{\text{free}}}{C_{\text{total}}}\times 100\%$$

where *C*_total_ is the total concentration of DE fed into micelles initially, and *C*_free_ is the concentration of DE not encapsulated in micelles.

In addition, the encapsulated amount of DE in DE-CMs was measured as follows:

(2) $$\text{Encapsulated}\;\text{amount}=(C_{\text{total}}-C_{\text{free}})\times V_{\text{sample}}$$

where *C*_total_ is the total concentration of DE fed into micelles initially, *C*_free_ is the concentration of DE not encapsulated in micelles, and *V*_sample_ is the volume of sample, 0.5 mL.

### Critical micelle concentration (CMC) determination

The CMC value of micelles was determined by a fluorescence probe, pyrene. (Kalyanasundaram & Thomas, 1977; Kim et al., 1999; Aguiar et al., 2003). A series of blank micellar solutions with concentrations ranging from 10^−4 ^g/L to 10^−1 ^g/L were mixed with the prepared pyrene films by an ultrasonic treatment until the final concentration of pyrene was 6 × 10^−7 ^mol/L. The mixture was incubated in water bath at 37 °C for 5 h and then kept away from light overnight. The fluorescence intensity was recorded by a fluorescence spectrophotometer (RF-5301PC, Shimadzu, Tokyo, Japan) at 391 nm (Xie et al., 2007). The CMC value of micelles was determined by a crossover point in the plot of the fluorescence intensity ratio of 338 to 336 nm (*I*_338_/*I*_336_) versus the logarithm concentration.

### Leakage studies of DE-CMs in varying media

Approximately 3 mL of DE-CMs was mixed separately with SGF and SIF respectively until the final volume of the solution was 75 mL. Then, the mixture was incubated with shaking at 37 °C for 2 h and oscillating at 100 rpm. Samples were treated as described in the Encapsulation efficiency (EE) of DE-CMs section to measure the free drug content in different media.

### *In vitro* precipitation studies

The *in vitro* precipitation studies were inspected by the paddle method using a dissolution tester (RCZ-2B6, Shanghai Huanghai, China) at 37 °C with rotation speed of 100 rpm. Briefly, the commercial formulation, DE-CMs and the mixture (DE, soluplus and TPGS) were first placed in 750 mL of SGF (pH 1.2) at 37 °C followed by stirring for 2 h. Next, 250 mL of sodium phosphate solution (0.2 mol/L) was quickly added to adjust the pH value of the medium to 6.8 and continually stirred for 3 h (Li et al., 2012). An aliquot of the 5 mL samples was withdrawn at the predetermined time intervals (15, 120, 150, 180, 240, and 300 min) with a replacement of an equal volume of temperature-equilibrated medium and subsequently filtered through a membrane filter (0.45 μm). The content of drugs was determined by UV spectrophotometry at 324 nm after demulsification.

(3) $$\text{Precipitation}\;\text{percentage}\;(\%)=(1-\frac{C^{\prime }}{C_{\text{total}}})\times 100\%$$

where *C′* is the content of the free drugs in the medium, and *C*_total_ is the total concentration of drugs initially fed into each group.

### Evaluation of cytotoxicity by 3-(4,5-dimethylthiazol-2-yl)-2,5-diphenyltetrazolium bromide (MTT) assay

Cytotoxicity of DE, dabigatran etexilate-loaded single micelles (DE-SMs) and DE-CMs was investigated by the MTT assay (Zhai et al., 2013). Caco-2 cells were supplied by KeyGEN BioTECH Co., Ltd. (Nanjing, China) and maintained in medium composite of DMEM (high glucose), 20% FBS and 1% penicillin/streptomycin solution. Cells were cultured at a density of 5 × 10^4^ cells/well in 96-well plates and incubated at 37 °C under 5% CO_2_ for 24 h. Then, the complete medium was removed and cells were further incubated with 100 μL of complete medium containing DE, DE-SMs and DE-CMs at 37 °C for 24 h, respectively. Next, the medium was replaced with 20 μL of MTT (5 mg/mL) solution and 80 μL of fresh medium. Following 4 h of incubation, the medium containing MTT was discarded and 150 μL of DMSO was added to each well to dissolve the granular crystallization. The optical density of the samples was measured at 490 nm wavelength using a microplate ELISA analyzer (Thermo Scientific, USA) (Fan et al., 2017a). The negative control was the group of Caco-2 cells without drugs. Cell viability was calculated as follows:

(4) $$\text{Cell}\;\text{viability}\;(\%)=(1-\frac{{\text{OD}}_{\text{negtive}\;\text{control}}-{\text{OD}}_{\text{sample}}}{{\text{OD}}_{\text{negtive}\;\text{control}}})\times 100\%$$

where OD_negative control_ is the optical density of the negative control group, and OD_sample_ is the optical density of the experimental group.

### Cellular transport studies

The Caco-2 cell monolayer is the most widespread model for cellular transport experiments to evaluate the efflux effect of P-gp (Hunter et al., 1993). The transepithelial electrical resistance (TEER) value of Caco-2 cell monolayer model was measured using a cell resistance meter (Millicell-ERS, Millipore, USA), and models were used for transport experiments when the TEER value exceeded 300 Ω·cm^−2^ (Andrew & Cameron, 2012).

For apical to basolateral (AP > BL) (basolateral to apical (BL > AP)) transport experiments, 1.5 mL of Hank’s Balanced Salt Solution (HBSS) loaded with DE, DE-SMs and DE-CMs (the concentration of DE in different groups was 200 μg/mL) was added to the AP side (BL side) and 2.6 mL of blank HBSS was added to the BL side (AP side), respectively. At fixed time intervals, 400 μL aliquots were taken from the BL side (AP side) with replacement of the fresh HBSS. The samples were frozen in a refrigerator at –80 °C until HPLC analysis. The HPLC method employed an ODS C18 column (250 × 4.6 mm, 5 μm, Dikma Technologies, China) and a methanol-water (75:25, v/v) mobile phase with a flow rate of 1.0 mL/min. The detection wavelength was 316 nm and the injection volume was 20 μL. The apparent permeability coefficient (Papp) and efflux ratio (ER) were calculated as follows:

(5) $$\text{Papp}=\frac{\mathrm{dQ/dt}}{A\times C_{0}}$$

where *dQ*/*dt* is the steady-state appearance rate of DE on the receiver side, *C*_0_ is the initial concentration of DE on the supply side, and *A* is the surface area of the cell monolayer.

(6) $$\text{ER}=\frac{{\text{Papp}}_{(\text{BL}\to \text{AP})}}{{\text{Papp}}_{(\text{AP}\to \text{BL})}}$$

where Papp_(BL→AP)_ is the Papp value of DE transferred from the BL side to AP side, and Papp_(AP→BL)_ is the Papp value of DE transferred from the AP side to BL side.

### Pharmacokinetics studies in rats

For the pharmacokinetic study, twelve male Sprague-Dawley rats were randomly divided into two groups (*n* = 6), fasted overnight but with free access to water before experiments. The commercial formulation and DE-CMs were orally administered to two groups at an equivalent dose of 30 mg/kg. An aliquot of 400 μL of blood samples was collected from the retro-orbital plexus of rats at predetermined times (0.25, 0.5, 0.75, 1, 1.5, 2, 2.5, 3, 4, 6, 8 and 12 h) and then centrifuged at 7000 rpm for 10 min to obtain plasma. Plasma sample (100 μL) was next mixed with 400 μL of methanol and 10 μL of the internal standard solution (theophylline, 2 mg/mL). The mixture was vortexed vigorously for 2 min and centrifuged at 7000 rpm for 10 min, then 400 μL of the supernatant was evaporated under nitrogen to obtain the residue. The residue dissolved in 100 μL of the mobile phase was further vortexed for 3 min and then centrifuged at 12,000 rpm for 10 min. Finally, 20 μL of the supernatant was analyzed by the HPLC method. The HPLC system was the same as that in the Cellular transport studies section except that ammonium formate-methanol (77:23, v/v) was employed as the mobile phase and the wavelength was set at 303 nm.

### Statistical analysis

Pharmacokinetic analysis was performed by PKSolver statistical software and all data are expressed as the mean ± SD. PKSolver is a fast and easy-to-use tool with a range of modules for routine and basic pharmacokinetic and pharmacodynamic analysis, and it can be directly accessed in an Excel spreadsheet (Zhang et al., 2010). Statistical analysis was performed using ANOVA of SPSS version 19.0 software and a Student’s *t*-test was conducted to evaluate the difference between groups. In all cases, *p <* .05 was considered statistically significant.

### Pharmacodynamics studies in rats

Eighteen male Sprague-Dawley rats were randomly divided into three groups, fasted overnight but had free access to water before experiments. Rats were orally administered the commercial formulation, DE-CMs and normal saline at a dose of 30 mg/kg, respectively. Blood samples were collected from the ophthalmic venous plexus of rats at the designed time intervals (0.25, 0.5, 0.75, 1, 1.5, 2, 2.5, 3, 4, 6 and 8 h) after administration. The activated partial thromboplastin time (APTT) was employed to analyze the anticoagulant effect of DE-CMs, and this value was measured using a commercially available kit (Nanjing Herb Source Bio-Tech, China) (Stangier, 2008; Andreas et al., 2012) containing sodium citrate anticoagulant (109 mM), kaolin-kephalin solution and CaCl_2_ solution (25 mM). Briefly, blood samples were mixed with 109 mM sodium citrate in a proportion of 9:1 and then centrifuged at 4000 rpm for 15 min. The supernatant was kept and kaolin-kephalin solution was balanced to room temperature. Next, 100 μL of supernatant was mixed with 100 μL of kaolin-kephalin solution, and the mixture was incubated at 37 °C for 5 min followed by 100 μL CaCl_2_ solution (25 mM, 37 °C) was added in. The mixture was further incubated in water bath at 37 °C for 30 s with continuous shake, and the time when fibrin filament exists was recorded.

## Results

### Preparation of DE-CMs and formulation screening

**Figure 1.**
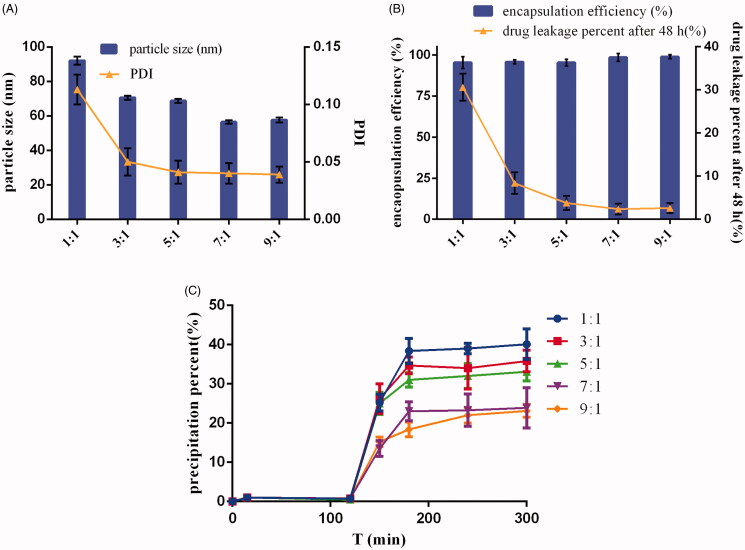
Effects of the ratio of soluplus and TPGS on the (A) particle size and PDI, (B) encapsulation efficiency and drug leakage percent, (C) precipitation in simulated gastrointestinal tract of DE-CMs (*n* = 3). PDI: polydispersity index; DE-CMs: dabigatran etexilate-loaded composite micelles.

Micelles have been used as promising nanosystems for drugs with low bioavailability due to their excellent solubilization of insoluble drugs, favorable particle sizes (10–100 nm) to penetrate cell membranes and the possibility for functionalization with different materials (Zhou et al., 2016a). However, drug leakage could occur after micelles are diluted by the body fluid. Furthermore, for weakly basic drugs, part of the leaked drugs from micelles could later precipitate in the intestinal tract, resulting in the poor drug absorption (Rajesh et al., 2006). Therefore, an ideal micelle system should decrease the percentage of drug leakage and drug precipitation *in vivo*. According to Figure 1(A,B), DE-CMs had a uniform size distribution (PDI < 0.15) and high encapsulation efficiency (>90%) at varying ratios of soluplus and TPGS (1:1, 3:1, 5:1, 7:1, and 9:1). Figure 1(A) also shows that an increase in the proportion of soluplus led to a decrease in particle size of micelles, and a minor particle size (56.47 ± 1.03 nm) was observed when the ratio of soluplus and TPGS was 7:1. In addition, the drug leakage percentage of DE-CMs decreased dramatically from 80.42 ± 8.73% to 6.02 ± 3.01% when the ratio of soluplus and TPGS increased from 1:1 to 7:1 (Figure 1(B)). The decrease of particle size did not result in the increase of drug leakage, and the reason for the decreased drug leakage of micelles could be as follows. TPGS, a representative polymer used in micelles, has a relatively high CMC (0.02 wt %), which leads to the formation of unstable micelles (the percent of DE leaked from single micelles composed with TPGS was 88.85 ± 3.34%) (Zhao et al., 2017). Soluplus as a newly graft copolymer with a very low CMC (0.0076 mg/mL) could form the relative stable micelles (the percent of DE leaked from single micelles composed with soluplus was 35.68 ± 0.93%) and decrease CMC value of micelles after mixing with TPGS. Thus, the stability of micelles was improved and the drug leakage decreased with an increase of soluplus content in formulation (Zhou et al., 2016b). Figure 1(C) indicates that the formation of drug precipitation in SIF was obviously inhibited when the ratios of soluplus and TPGS were 7:1 and 9:1, and the precipitation percentage of DE showed no significant difference at ratio of 7:1 and 9:1. Finally, in order to make inhibition of TPGS on P-gp efflux more effective, the proportion of TPGS in formulation was increased and the optimum formulation of DE-CMs was found at ratio of soluplus and TPGS of 7:1.

### Characterization of DE-CMs and DE-SMsV

**Figure 2.**
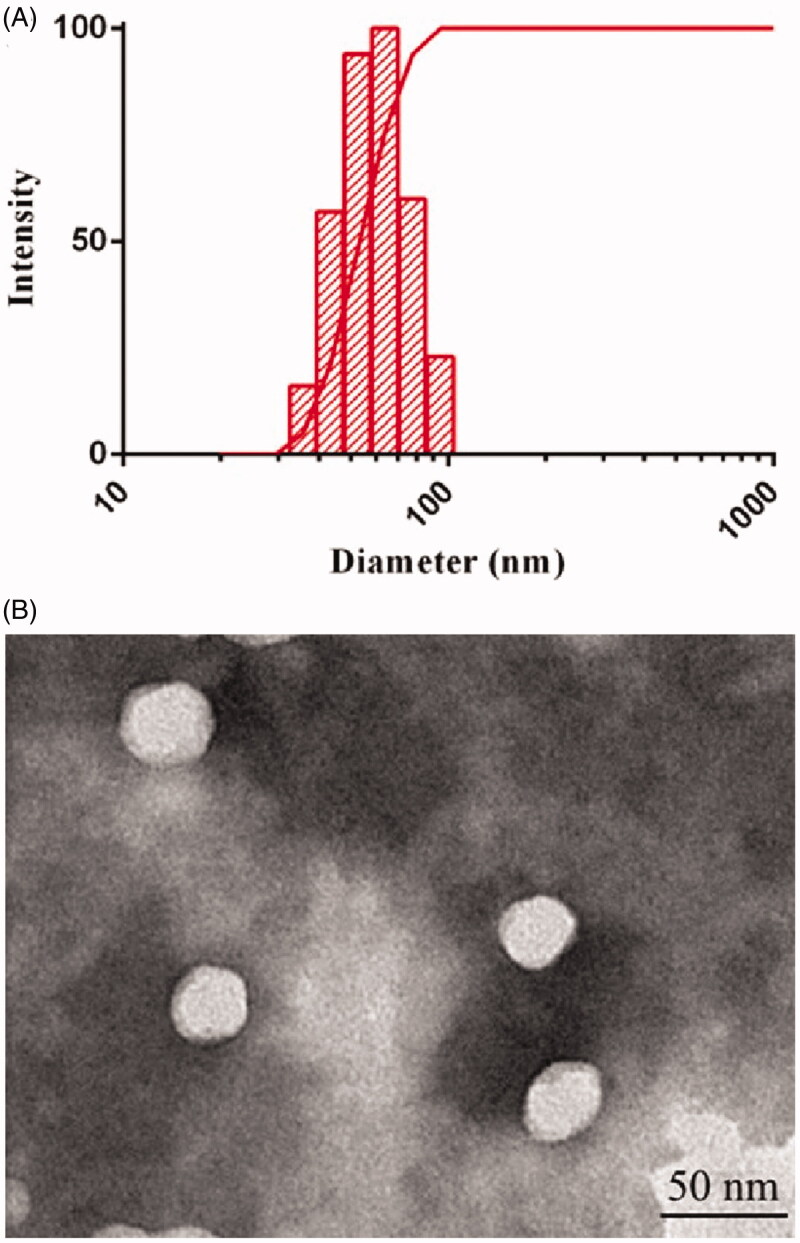
(A) Particle size distribution and (B) TEM image of DE-CMs. TEM: transmission electron microscope; DE-CMs: dabigatran etexilate-loaded composite micelles.

As shown in Figure 2(A), the mean particle size of DE-CMs was 57.5 ± 0.8 nm and the PDI was 0.054 ± 0.017. The surface charge of DE-CMs was neutral with zeta potential of 1.24 ± 1.23 mV, indicating that DE-CMs might directly pass through the mucus layer of GI tract for transmembrane transport and absorption (Lai et al., 2007). In addition, the particle size and zeta potential of DE-SMs was 51.96 ± 6.25 nm and –2.12 ± 0.94 mV, respectively, indicating that the addition of TPGS had no obvious effect on the particle size and surface charge of micelles. Figure 2(B) shows the TEM image of DE-CMs. Obviously, DE-CMs displayed a homogeneous and spherical morphology with the particle size of approximately 40 nm. The reason for the difference in particle size measured by TEM and DLS is that the size of DE-CMs measured by DLS was the hydration radius, while DE-CMs measured by TEM was dry without the hydration layer (Fan et al., 2017b).

**Figure 3.**
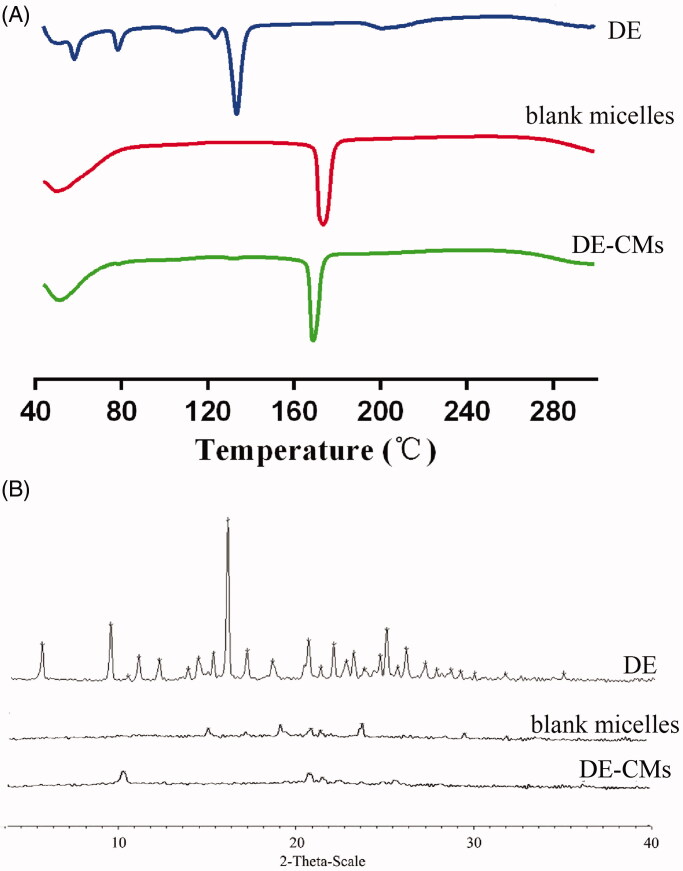
(A) DSC analyses and (B) XRD analyses of DE, blank composite micelles and DE-CMs. DSC: differential scanning calorimetry; XRD: X-ray powder diffractometry; DE: dabigatran etexilate; DE-CMs: dabigatran etexilate-loaded composite micelles.

Figure 3(A) shows that DE exhibited a principle endothermic peak at 129.2 °C and several small peaks at 55.7, 76.1 and 119.8 °C, respectively, indicating that DE was a drug with polymorphic form. However, the endothermic peaks of DE did not exist in the thermogram of DE-CMs, which might be attributed to the encapsulation of DE in DE-CMs in the amorphous form, rather than absorbed on the surfaces of DE-CMs. In diffraction patterns shown in Figure 3(B), intensive crystalline peaks of DE were found at 8.790, 15.488 and 24.610°, and there is no characteristic peak in the diffraction pattern of blank micelles and DE-CMs. The disappearance of crystalline peaks of DE in the diffraction pattern of DE-CMs further confirmed that DE was encapsulated into composite micelles at an amorphous state (Yuan et al., 2018), indicating the formation of DE-CMs.

### Determination of CMC

One of the major requirements for the formation of micelles is that the concentration of copolymers should exceed the CMC, and micelles with a low CMC can more easily retain their integrity after dilution by body fluid (Letchford & Burt, 2007). Thus, CMC is an important parameter to evaluate the stability of micelles. Pyrene, a hydrophobic fluorescent probe, prefers to move into the hydrophobic cores of micelles from the medium, resulting in changes of the polarity of the microenvironment around it. At that point, a mutation in the ratio of fluorescence intensity of pyrene at 338 nm and 336 nm will exist (Zhai et al., 2013; Dou et al., 2014). Therefore, pyrene is commonly applied to determine the CMC value.

**Figure 4.**
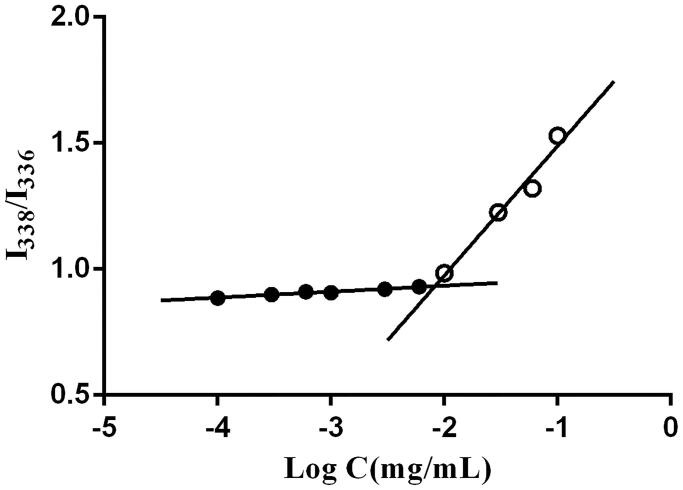
*I*_338_/*I*_336_ versus log *C* of DE-CMs. DE-CMs, dabigatran etexilate-loaded composite micelles.

As shown in Figure 4, CMC was defined as the concentration corresponding to the crossover point of the horizontal tangent and the slope tangent to the curve (the curve of the fluorescence intensity ratio of 338 to 336 nm versus the logarithm concentration was not shown in Figure 4). The CMC of composite micelles was as low as 0.0083 mg/mL, suggested that micelles presented a strong dilution-proof ability and good stability.

### Leakage studies on DE-CMs in varying media

**Figure 5.**
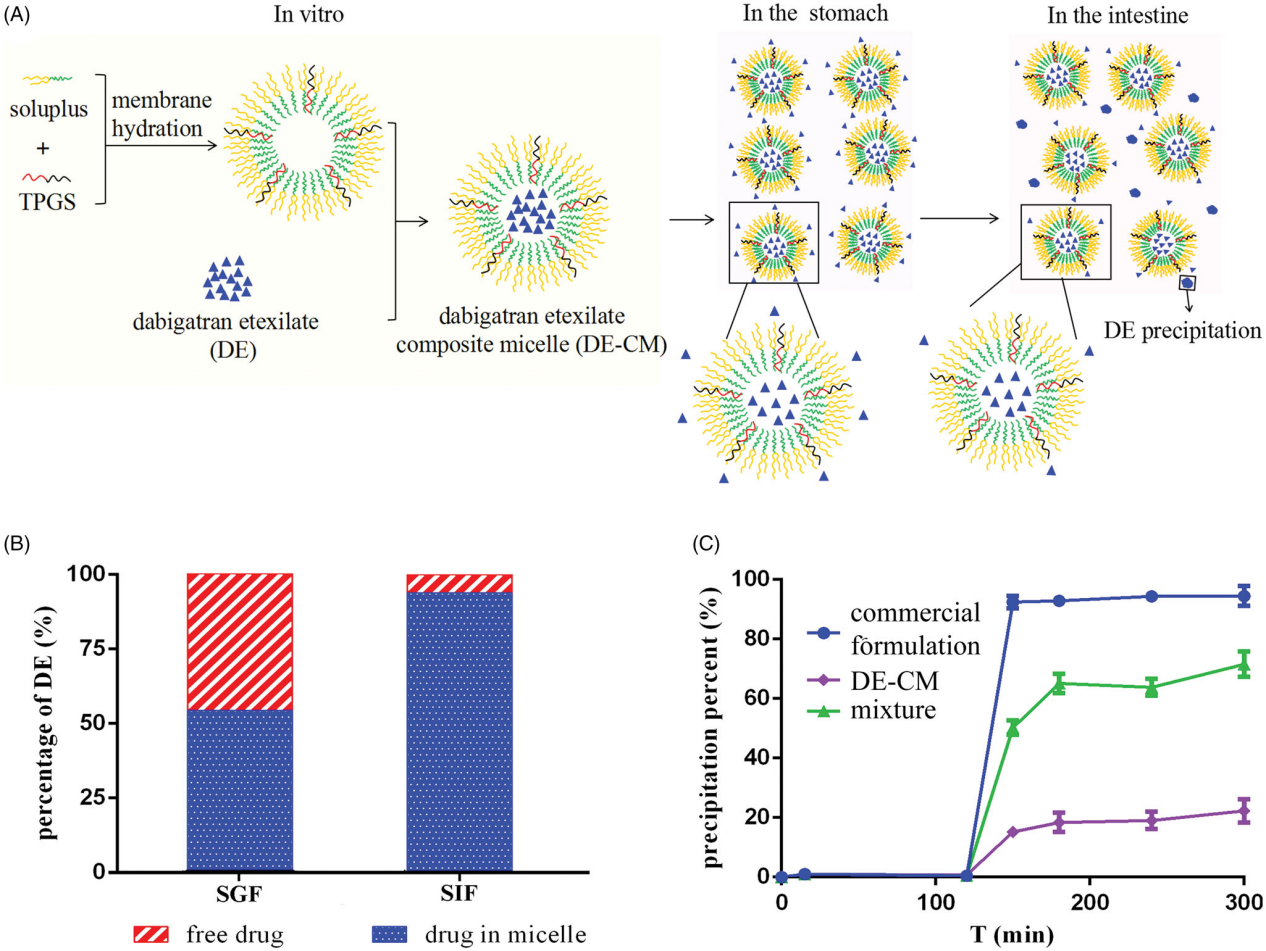
(A) *In vitro* formation and transport process of DE-CMs in the GI tract. (B) Percentage of free drugs and drugs in DE-CMs after incubation in SGF, SIF and distilled water under 37 °C for 2 h. (C) *In vitro* precipitation of the commercial formulation, DE-CMs, mixture (DE, soluplus and TPGS) under SGIF (*n* = 3). DE-CMs: dabigatran etexilate-loaded composite micelles; SGF: simulated gastric fluid; SIF: simulated intestinal fluid; SGIF: simulated gastrointestinal fluid.

To mimic the leakage of DE from DE-CMs in the GI tract, leakage studies were conducted in media at different pH values. As presented in Figure 5(B), the percentage of DE leaked from DE-CMs decreased from 45.98 ± 2.61% in SGF to 7.95 ± 2.02% in SIF with increasing pH value. This demonstrated that DE preferred to leak from micelles in an acidic environment, which was attributed to its high solubility in SGF. Moreover, with the commercial formulation as a comparison which almost completely dissolved in SGF, only approximately 50% of DE leaked from DE-CMs and dissolved in SGF, suggesting that DE-CMs could effectively reduce drug leakage in the stomach. It is probably because the affinity between DE and soluplus was increased due to the interaction of non-polar hydrocarbon chain of the drug and hydrophobic chain of soluplus, thus making drugs prefer to be encapsulated into the micelles and then the leakage of the drug was inhibited (Punčochová et al., 2016).

### *In vitro* precipitation studies

In our previous studies, we found that the solubility of DE is high (19.245 mg/mL at 37 °C) in SGF and very low (3.060 × 10^−3 ^mg/mL at 37 °C) in SIF (Chai et al., 2016). Thus, DE which leaked from micelles in the stomach was likely to precipitate when moving to the intestine owing to the sharp decrease of drug solubility (Figure 5(A)). To mimic the actual conditions of DE in the GI tract, *in vitro* precipitation studies were conducted in the simulated gastrointestinal fluid. As Figure 5(C) shows, DE in all formulations dissolved completely in SGF for the first two hours. However, the precipitation percent of DE in DE-CMs (22.25 ± 3.88%) produced an obvious decrease compared with that of the commercial formulation (93.98 ± 1.28%) after the medium was converted to SIF, indicating that DE-CMs significantly inhibited drug precipitation in the GI tract (*p <* .01). In addition, the solubility of DE-CMs was increased to 0.0159 mg/mL in SIF, which further confirmed the effects of composite micelles on the improved drug solubility and the decreased drug precipitation in SIF.

It is also worth noting that the precipitation percent of DE in the mixture (DE, soluplus and TPGS) in SIF was lower than that of the commercial formulation, which implied that carrier materials used in micelles had a certain solubilizing effect on DE. It might be attributed to the amino groups in DE and hydroxyl groups in soluplus forming intermolecular hydrogen bonds (Anby et al., 2012), which were beneficial to form fluid interfacial layers on surfaces of the crystal nucleus and these layers could further inhibit drug precipitation. Additionally, the percentage of DE leaked from DE-CMs in SGF was 45.98 ± 2.61%, while the precipitation percentage of DE in DE-CMs decreased to 22.25 ± 3.88%. This result demonstrated that some of the leaked DE were solubilized and did not precipitate completely when the medium was adjusted to SIF, which is probably because DE leaked from DE-CMs was again encapsulated in micelles or because part of the leaked DE was solubilized through hydrogen-bonding interactions between DE and soluplus.

In summary, with DE-SMs and the commercial formulation as comparisons, *in vitro* precipitation studies showed that DE-CMs obviously inhibited the precipitation of DE in the GI tract. It might be attributed to the following reasons: (1) DE was encapsulated in micelles, which inhibited drug leakage in SGF and subsequently resulted in the decreased drug precipitation in SIF. (2) Part of the leaked DE in SGF was solubilized by the carrier materials.

### Evaluation of cytotoxicity by MTT assays

**Figure 6.**
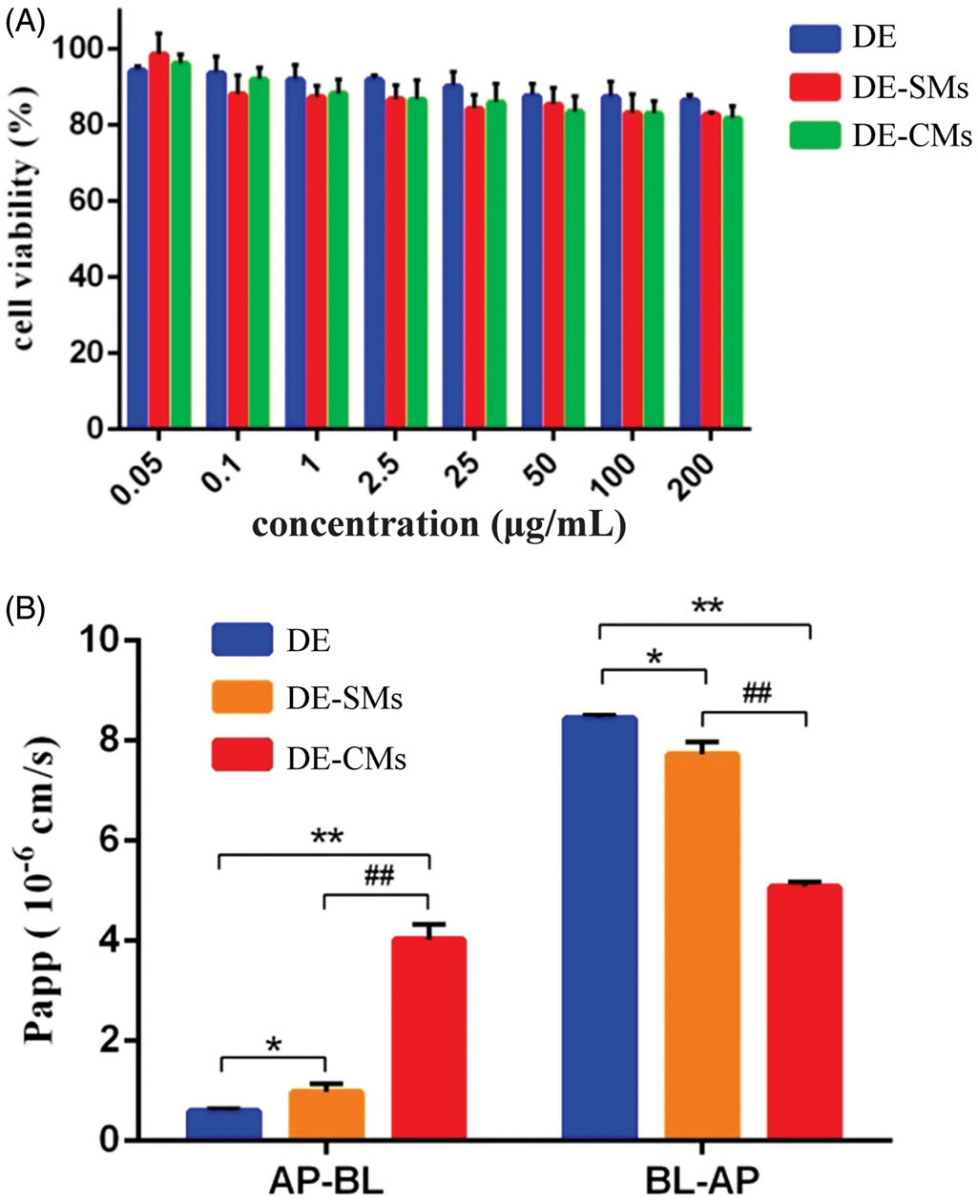
(A) Cell viability of DE, DE-SMs and DE-CMs on Caco-2 cells (*n* = 3). (B) Permeability of DE, DE-SMs and DE-CMs across Caco-2 cell monolayer (*n* = 3). *p < .05, **p < .01 versus DE; #*p <* .05, ##*p <* .01 versus DE-SMs. DE: dabigatran etexilate; DE-SMs: dabigatran etexilate-loaded single micelles; DE-CMs: dabigatran etexilate-loaded composite micelles; AP-BL: from apical to basolateral; BL-AP: from basolateral to apical.

An MTT assay was conducted to determine the safety concentration range of different formulations for the following cellular experiments. The results are exhibited in Figure 6(A). The cytotoxicity of different formulations presented a concentration-dependent property and the cell viability was negatively correlated with the tested concentration. Furthermore, the cell viability of DE, DE-SMs and DE-CMs was all higher than 80% with the tested concentrations ranging from 0.05 μg/mL to 200 μg/mL. Such results implied that DE, DE-SMs and DE-CMs had good biocompatibility at this concentration range (Ran et al., 2011).

### Cellular transport studies

The TEER value of the Caco-2 cell monolayer reached 339.29 ± 29.66 Ω·cm^−2^ after 21 days of incubation, indicating that the cell models were successfully established with a dense monolayer structure.

According to the FDA guidelines, if the ER value of a drug is greater than 2, then this drug is likely to be a P-gp substrate (Ishiguro et al., 2014). In addition, a decrease of the ER value is expected after the P-gp substrate is mixed with P-gp inhibitors (Wang et al., 2003). Hence, the ER value is commonly used to evaluate the effect of P-gp inhibitors. As shown in Figure 6(B), the Papp values of DE across the Caco-2 monolayer were 0.59 ± 0.055 × 10^−6 ^cm/s (AP to BL) and 8.44 ± 0.072 × 10^−6 ^cm/s (BL to AP), resulting in an ER value of 14.23. This confirmed that DE was a P-gp substrate, which could be pumped out from enterocytes to the lumen after oral administration. Compared with DE, DE-SMs exhibited a significant increase (*p < .05) in Papp _(AP to BL)_ from 0.59 ± 0.055 × 10^−6 ^cm/s to 0.97 ± 0.17 × 10^−6 ^cm/s and a significant decrease (*p < .05) in Papp_(BL to AP)_ from 8.44 ± 0.072 × 10^−6 ^cm/s to 7.72 ± 0.26 × 10^−6 ^cm/s, respectively. Consequently, the ER value of DE-SMs was 7.96, which was 1.79-fold lower than that of DE, indicating that DE-SMs inhibited the efflux effect of P-gp. The reason might be that drug-loaded micelles with small particle sizes (<100 nm) could cross the biological membrane by endocytosis (Mathot et al., 2007), which prevented drugs from being recognized by P-gp and thus inhibited the efflux effect of P-gp. Furthermore, the Papp_(AP to BL)_ and the Papp_(BL to AP)_ of DE-CMs were 4.01 ± 0.32 × 10^−6 ^cm/s and 5.07 ± 0.11 × 10^−6 ^cm/s, respectively, resulting in an ER value for DE-CMs of 1.27, which was 6.27-fold and 11.20-fold lower than that of DE and DE-SMs, respectively. Therefore, DE-CMs with the addition of TPGS greatly decreased the ER value and exhibited the stronger inhibiting effect on the P-gp efflux of DE compared with DE-SMs and DE. This result could be attributed to two reasons: (1) TPGS inhibited the efflux effect of P-gp by influencing the activity of P-gp ATPase, resulting in a decrease of the ER value (Collnot et al., 2007). (2) Micelles could move into cells by endocytosis and DE encapsulated in DE-CMs would not be recognized by P-gp, thus, reducing the interactions between DE and P-gp (Diao et al., 2009).

### Pharmacokinetics studies

**Figure 7.**
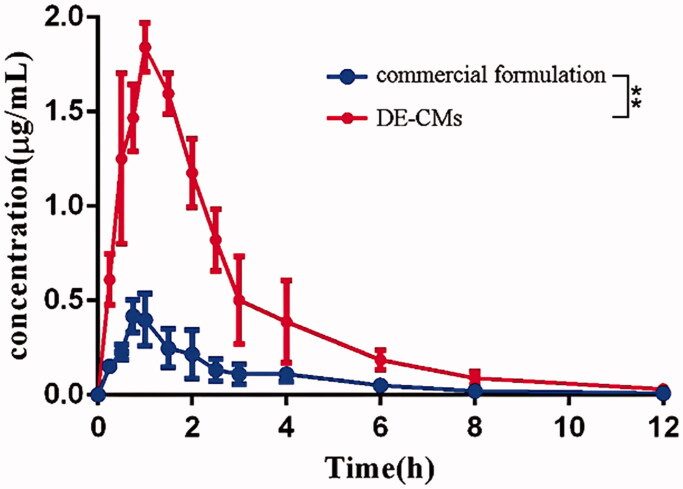
Dabigatran plasma concentration-time of the commercial formulation and DE-CMs in rats (*n* = 6). **p < .01 versus the commercial formulation. DE-CMs: dabigatran etexilate-loaded composite micelles.

The plasma concentration-time profiles for the commercial formulation and DE-CMs are illustrated in Figure 7. The main pharmacokinetic parameters are listed in Table 1. The peak plasma concentration (*C*_max_) of the commercial formulation (0.4296 ± 0.0898 μg/mL) and DE-CMs (1.850 ± 0.115 μg/mL) were obtained at 0.9167 ± 0.1443 h and 1.167 ± 0.289 h after oral administration, respectively, indicating that DE-CMs produced an obvious improvement of oral absorption *in vivo*. The AUC_0-t_ treated with DE-CMs was 4.61-fold higher than that of the commercial formulation. Additionally, the relative bioavailability of DE-CMs was 456.58% with the commercial formulation as a reference, which revealed that the DE-CMs effectively improved the oral bioavailability of DE in rats.

**Table 1.** Pharmacokinetic parameters of dabigatran etexilate in rats after oral administration of commercial formulation and DE-CMs (mean ± SD, *n* = 6).

Parameters	Unit	Commercial formulation	DE-BMs
*C*_max_	μg/mL	0.4296 ± 0.0898	1.850 ± 0.115**
*T*_max_	h	0.9167 ± 0.1443	1.167 ± 0.289
*t*_1/2_	h	2.336 ± 0.544	2.256 ± 0.519
AUC_0–t_	μg/mL·h	1.079 ± 0.330	4.975 ± 0.733**
AUC_0–∞_	μg/mL·h	1.112 ± 0.340	5.076 ± 0.710**
Relative bioavailability	%	–	456.58

*C*_max_, peak concentration; *T*_max_, time to peak concentration; *t*_1/2_, elimination half-life; AUC, area under curve; DE-CMs, dabigatran etexilate-loaded composite micelles.

*p < .05, **p < .01 versus the commercial formulation.

### Pharmacodynamics studies

**Figure 8.**
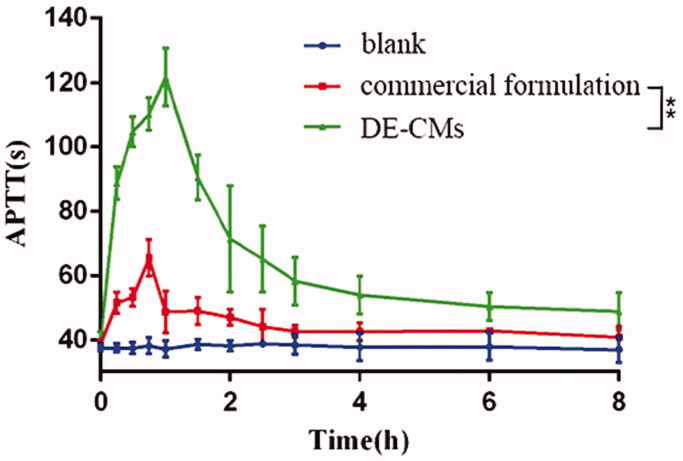
Time curves of APTT after oral administration of commercial formulation and DE-CMs (*n* = 6). **p < .01 versus the commercial formulation. APTT: activated partial thromboplastin time; DE-CMs: dabigatran etexilate-loaded composite micelles.

The APTT assay, targeting the intrinsic pathway of the coagulation cascade, is often used to assess the antithrombotic and anticoagulant effects of dabigatran in rats (Wienen et al., 2007). Compared with the APTT of rats receiving normal saline without obvious fluctuation, the APTT of rats receiving DE-CMs and the commercial formulation were both prolonged, illustrating that both of them had anticoagulant activity (Figure 8). In addition, the APTT of DE-CMs was higher than that of the commercial formulation at each time point, and the peak value of DE-CMs was 121.62 ± 9.07 s, which was significantly prolonged over 1.86-fold compared with the commercial formulation a peak value of 65.47 ± 5.61 s (*p <* .01). This result demonstrated that the DE-CMs obviously improved the anticoagulant activity of DE *in vivo* compared with the commercial formulation.

## Conclusion

DE, a novel anticoagulant drug, has been granted approval by the European Medicines Agency for the treatment of thromboembolic disease following elective total hip or knee replacement surgery and has been marketed by the US Food and Drug Administration for the prevention in patients with nonvalvular atrial fibrillation (Bellamy et al., 2009; Wilcox et al., 2011; Greig & Mckeage, 2014). However, the oral bioavailability of DE is very low (approximately 3–7%), and this greatly restricted its clinical applications (Wang et al., 2013). In this study, DE-CMs with a small particle size and low CMC were successfully prepared here and were confirmed to be responsible for the decreased drug precipitation and the inhibited P-gp efflux in the GI tract. As a result, significant improvements in the oral bioavailability of DE were observed as suggested by the pharmacokinetic studies of DE-CMs, and the enhanced anticoagulation activity *in vivo* was also displayed in the pharmacodynamic studies compared with the commercial formulation. Hence, the developed composite nanaocarriers may have applications as promising systems to decrease the precipitation and improve the therapeutic effect of weakly basic drugs.
